# Description of patients with eating disorders by general practitioners: a cohort study and focus on co-management with depression

**DOI:** 10.1186/s40337-023-00901-0

**Published:** 2023-10-19

**Authors:** Jean Sébastien Cadwallader, Massimiliano Orri, Caroline Barry, Bruno Falissard, Christine Hassler, Caroline Huas

**Affiliations:** 1 School of Medicine, Department of General Practice, Sorbonne Université, Paris, France; 2 INSERM, Institut Pierre Louis d’Epidémiologie et de Santé Publique, Sorbonne Université, Paris, France; 3 INSERM, UMR 1018, Centre de Recherche en Épidémiologie et Santé des Populations (CESP), Paris-Saclay University, Paul Brousse Hospital, Villejuif Cedex, France; 4 McGill Group for Suicide Studies, Department of Psychiatry, Douglas Mental Health University Institute, McGill University, Montréal, QC Canada; 5 Bordeaux Population Health Research Centre, INSERM U1219, University of Bordeaux, Bordeaux, France; 6 Fondation Santé des Etudiants de France (FSEF), Service Hospitalo-Universitaire de Santé Mentale de l’Adolescent et du Jeune Adulte (SAMAJA), Paris, France; 7 Department of Family Medicine, Faculty of Health Sciences Simone Veil, University Versailles-Saint-Quentin-en-Yvelines (UVSQ), 78180 Montigny le Bretonneux, France

**Keywords:** General practice, Cohort studies, Feeding and eating disorders, Depression, Disease management, Patient-centered care

## Abstract

**Background:**

International guidelines often state that general practitioners (GPs) provide early management for most patients with eating disorders (EDs). GP management of EDs has not been studied in France. Depressive disorders are often a comorbidity of EDs. The aims of this study were to describe in France the characteristics of people with all subcategories of EDs (Anorexia Nervosa, Bulimia Nervosa, ED Not Otherwise Specified) managed by their GPs and to study the management temporality between depression and all subcategories of EDs.

**Methods:**

Retrospective cohort study of patients with EDs visiting French GPs. Data collected from 1994 through 2009 were extracted from the French society of general electronic health record. A descriptive analysis of the population focused on depression, medication such as antidepressants and anxiolytics, and the management temporality between depression and EDs.

**Results:**

1310 patients aged 8 years or older were seen at least once for an ED by a GP participating in the database out of 355,848 patients, with a prevalence rate of 0.3%. They had a mean age of 35.19 years, 82.67% were women. 41.6% had anorexia nervosa, 26.4% bulimia nervosa, and 32% an ED not otherwise specified. Overall, 32.3% had been managed at least once for depression, and 18.4% had been prescribed an antidepressant of any type at least once. Benzodiazepines had been prescribed at least once for 73.9% of the patients treated for depression. Patients with an ED seen regularly by their GP (“during” profile) received care for depression more frequently than those with other profiles. 60.9% had a single visit with the participating GP for their ED Treatment and management for depression did not precede care for EDs.

**Conclusions:**

Data extracted from the French society of general practice were the only one available in France in primary care about EDs and our study was the only one on this topic. The frequency of visits for EDs was very low in our general practice-based sample. Depressive disorders were a frequent comorbidity of EDs. GPs could manage common early signs of depression and EDs, especially if they improved their communication skills and developed collaborative professional management.

## Abstract (plain language summary)

International guidelines often state that general practitioners (GPs) provide early management for most patients with eating disorders (EDs). Depressive disorders are often a comorbidity of EDs. The aims of this study were to describe in France the characteristics of people with all subcategories of EDs managed by their GPs and to study the management temporality between depression and all subcategories of EDs. We carried out a cohort study with the only French database available in general practice. 1310 patients aged 8 years or older were seen at least once for an ED by a GP participating in the database out of 355,848 patients. They had a mean age of 35.19 years, 82.67% were women. 41.6% had anorexia nervosa, 26.4% bulimia nervosa, and 32% an ED not otherwise specified. 32.3% had been managed at least once for depression. Benzodiazepines had been prescribed at least once for 73.9% of the patients treated for depression. Management for depression did not precede care for EDs. The frequency of visits for EDs was very low in our general practice-based sample. GPs could manage common early signs of depression and EDs, especially if they improved their communication skills and developed collaborative professional management.

## Background

Eating disorders (EDs) such as anorexia nervosa (AN) or bulimia nervosa (BN) have severe consequences [1–3]. AN has the highest mortality rate of all psychiatric disorders [1], BN is also associated with elevated mortality [4], nearly 3 times higher in patients with BN than controls [5]. Both disorders are associated with high rates of suicidality [3, 6, 7] and both psychiatric [8, 9] and somatic comorbidities, such as depression, anxiety, drug and alcohol addiction, gastrointestinal symptoms, and dental problems due to vomiting [10, 11]. They are also associated with social impairment [12–14]. Most psychiatric disorders occur during the adolescence, with more than 50% before the age of 14 years old [15]. That is why early detection is a challenge for mental health to avoid severe complications and individual burdens for young adults [15]. GPs are often considered in the secondary care and mental health care literature as the main actors for early detection, prevention and management of ED patients [16]. In the general population, incidence rates are between 4.2 and 7.7 per 100,000 person-years for AN and 6.1 to 12.2 per 100,000 person-years for BN. In general practice or primary care settings, incidence rates for diagnosed EDs have been as much as 20 times lower than in studies in the general population [17], This may be due to problems with detection or because patients with EDs may avoid GP visits [18, 19] although they have been shown to visit GPs more frequently than controls in the 5 years before diagnosis [20]. Community studies reported that fewer than one in ten cases of BN or BED and fewer than half the cases of AN and subclinical AN are detected [21, 22]. This finding indicates that GPs have opportunities for early prevention, early detection and management of EDs [16]. To our knowledge, no studies of early diagnosis have taken place in GP in France, even though some studies have been carried in the UK [17] or the Netherlands [23]. All the data available in France is about patients hospitalized for EDs.

It has often been hypothesized in the literature that depression precedes EDs [24]. The lifetime prevalence of depression in patients with EDs ranges from 30 to 40% [25]. Identification of depression in GP or primary care might be one way to detect early ED symptoms to manage the major consequences earlier described.

The aims of this study were to describe in France the characteristics of people with all subcategories of EDs (Anorexia Nervosa, Bulimia Nervosa, ED Not Otherwise Specified) managed by their GPs and to study the management temporality between depression and all subcategories of EDs.

## Methods

### General practitioner sampling

This cohort study used the only longitudinal French GP electronic health record belonging to the Société française de médecine Générale (French Society of General Practice), referred to SFMG-DB [26]. From 1993 through 2009, 112 GPs working mainly in solo practices routinely compiled data concerning their visits. These patient visits were largely representative of all French GP visits in terms of age, gender, and type and number of prescriptions for medication [27]. French patients visit their GPs with a mean of six visits or contacts per year in average and 2 to 3 reasons for consultation for one visit [27]. The French care system gives free access for GP visits. Routine clinical coding is very limited in France. In the SFMG-DB, diseases and related health problems are coded using the Dictionary of Consultation Results (DCR), which has been validated in France [28]. Transcoding between the DCR and the international classification of diseases (ICD) has also been previously validated [29]. GPs using DCR reported coding more systematically and expressed greater willingness to carry on coding on a routine basis compared with other classifications [29]. The database included 185,991 women and 169,857 men, aged 55.4 years in average, whose sociodemographic characteristics were not different than those of the individuals included in the French health insurance system database [27].

### Patients

This study included patients aged 8 years or older, as recommended by National Institute for Health and Care excellence in 2017 [30], seen at least once for an ED, according to the coding described below, by a GP participating in the database. The most recent classification of EDs in use at the time our data were collected was the DSM-4-TR [31]. Patients were considered included at the time of their first visit involving an ED with a GP contributing to the database. In all, 1,589 patients met these criteria. We excluded 126 because of missing data about the dates of their visits and 144 more with a secondary ED such as malnutrition, due to other diseases seen frequently in GP settings, cancer and HIV, at any time from before the inclusion to 5 years afterwards. We excluded nine patients because of missing data about gender. Finally, the study included 1,310 patients with at least one visit about an ED between 1994 and 2007 with a GP participating in the database.

### Codes for diseases and common comorbidities

In the SFMG-DB, multiple consultation results (CR) can occur during one visit [28]. The 1,310 patients included with at least one ED consultation result (ED CR) in the database were defined as "managed for an ED".

To achieve our second objective, we identified among the 1,310 ED patients included those with at least one consultation result of “depression”, a merger of the “Depression” and “Depressive mood” consultation results and defined them as managed for “depression." We also collected data on other diseases and common comorbidities such as gastrointestinal symptoms, gynecological symptoms, and dental problems [22, 32–35].

### Antidepressants and other medical prescriptions

In the SFMG-DB, prescriptions are coded according to the Anatomical Therapeutic Chemical (ATC) Classification System (WHO, 2006). We selected seven therapeutic categories potentially related to ED symptoms and common comorbidities detailed above: antidepressants; benzodiazepine (BZD)-derivative anxiolytics and BZD-related drugs, drugs used in addictive disorders; antacids; drugs for constipation; drugs for functional gastrointestinal problems; and diuretics.

### Data management and analysis

The ED consultation result was built with the subcategories reported by GPs during the inclusion visit, which was the first visit with an “eating disorder consultation result” (ED CR): AN, BN, or ED not otherwise specified (unclear or nonspecific) (EDNOS). We also studied the total durations of GP management, defined as the duration of time for which the patient was included in the database, and of specific management for ED or depression. Data extraction enabled us to study variables such as age, gender, dates of visits, total number of visits, number of visits specified with ED, and number of visits with depression.

#### Description of ED follow up profiles: inclusion of all patients with an ED visit in the database and a glance at GPs follow ups

We described different EDs follow up profiles based on the inclusion visit, which was the first visit with an ED CR. Data construction led to four profiles describing ED follow-up according to the chronological position of the inclusion visit. These four profiles were defined for ED follow-up: “first visit profile”, when the inclusion visit was the patient’s first contact ever with the GP of the database, “last visit profile” when it was the last contact with the GP, “during follow up profile “ when it occurred between the first and last visits, “unique visit profile” when it was the only contact.

#### Profiles of follow-ups for depression concerning patients with eating disorders taking into account the inclusion visit

We also built variables describing different chronological profiles of depression and ED management, by comparing the position of the inclusion visit relative to the period of depression management as 4 profiles: profile 1, when the depression management had started and finished before the inclusion visit, profile 2, when it started before the inclusion visit and finished simultaneously with the end of ED management, profile 3 when it started at or after the inclusion visit and finished with the end of ED management, profile 4, when it started and finished after ED care had ended.

#### Model of chronological descriptions between ED and depression

To achieve our second objective, we built a chronological descriptions model, exclusively for the patients with the “during” profile — those with visits both before and after their management for ED). The model was structured into three categories according to the order and timing between the first visit including management for depression (with a depression consultation result) and the inclusion visit for ED with an ED CR: exclusively before the inclusion visit for ED, the same day as the inclusion visit for ED, exclusively after the inclusion visit for ED.

Prescriptions for medications were finally described over the total duration of overall management for 546 patients. 764 were excluded because of missing data. These two populations did not differ for age, gender, or total duration of overall follow-up. We analyzed the data of patients aged 18 and older to compare them with other studies on adult patients of the same database and and also because medications guidelines before 18 were often different than those for adults, especially for antidepressants [30].

Data were described with adapted tests (chi-square, Student t-tests or ANOVA). The chronological descriptions model was analyzed with a global chi-square test for goodness of fit. The significance threshold was set at *p* < 0.05, without adjustment for multiple comparisons [36, 37]. Analyses were performed with SAS software and R 2.3.2 software. The variables we built were: subcategories of ED such as anorexia, bulimia, ED not otherwise specified; ED follow-up profiles with “first”, “last”, “during”, “only”; profiles of chronological descriptions between EDs and depression such as profile 1, 2, 3, 4.

### Ethics approval

The SFMG-DB was accredited by the National Research Registration committee with an ethics approval in 2006 (Comité National de l’Informatique et des Libertés-approval n°311668) and is publicly available.

## Results

### Description

Of the 355,848 patients seen by GPs in our data, 1,310 (0.36% of the database) met the inclusion criteria **(**Table 1**)**; 82.67% of them were women. According to the database, their mean age at the start of management was 35.19 years, 80.9% were older than 18 years, and 19.16% of the women were older than 50 years. The median number of visits for their overall duration of management was 17, the mean number of visits for ED was 2.97, and the mean total number of consultation results was 62.34. The total duration of overall management by GPs averaged 278.60 weeks, and that of ED management 20.45 weeks. Moreover, 5.6% of the patients with ED had anxiety, 8.4% gastrointestinal symptoms, 6.8% gynecological symptoms, and one person had dental issues.

**Table 1:** Description of patients with eating disorders (n = 1310) and according to ED subcategories

Subcategories of ED (n; % of total population)	Total sample (n = 1310, 100.00%)				AN (n = 545, 41.6%)				BN (n = 346; 26.4%)				EDNOS (n = 419; 32,0%)				Global comparison (anova or X square)/ results (*p*)
N (% women)	1083 (82.69%)				407 (74.68)				314 (90.75)				362 (86.39)				X2 = 44.13 *p* < 0.001
Value of central tendency	Mean	Standard deviation	Median	Range	Mean	Standard deviation	Median	Range	Mean	Standard deviation	Median	Range	Mean	Standard deviation	Median	Range	–
Age at first management for ED (years)	35.19	20.79	28.00	8–103	37.88	24.87	27.00	8–103	32.34	14.22	30.00	8–97	34.34	18.17	28.00	9–97	F = 102.58 p < 0.001
Total number of CR	62.34	127.7	24	1–1916	75.40	163.40	25.00	1–1916	48.16	63.77	27 00	1–558	58.11	108.40	20.25	1–1439	F = 5.33 p < 0.005
Total number of visits	31.66	41.20	17.00	1.362	34.86	48.77	17.00	1–362	28.80	29.54	19.00	1–132	30.75	39.00	15.25	1–243	F = 2.53 p = 0.08
Total duration of overall management (weeks)	278.60	223.10	245.50	0–785	280.70	222.70	264.00	0–785	302.50	227.30	275.50	0–775	256.43	217.23	215.25	0–762	F = 4.11 p < 0.05
Duration of ED follow-up (weeks)	20.45	62.98	0.00	0–677	14.36	40.32	0	0–445.70	16.19	45.21	0	0–295.9	31.78	91.78	0	0–667.1	F = 10.26 p < 0.05

*ED* Eating Disorders, *CR* Consultation Result, *AN* Anorexia Nervosa, *BN* Bulimia Nervosa, *EDNOS* Eating Disorders not otherwise specified

The GPs had diagnosed 41.6% of the 1,310 ED patients with (AN), 26.4% with (BN), and 32% with EDNOS (Table 1). The percentage of women with AN was lower than in other subcategories (74.68% vs. 90.75% for BN and 86.39% for EDNOS, *p* < 0.001). AN patients were also older (37.88 years vs. 32.34 for BN and 34.34 for EDNOS, *p* < 0.001) and had more total consultation results (75.40 vs. 48.16 for BN and 58.11 for EDNOS, *p* < 0.001) and a shorter duration of ED follow-up (14.36 vs 16.19 for BN and 31.78 for EDNOS, *p* < 0.05). BN patients had a longer duration of overall management (302.50 vs 280.70 for AN and 256.43 for EDNOS, *p* < 0.05) **(**Table 1).

### ED management

The inclusion visit with a GP participating in the database was the first visit ever for ED for 67.1% of the patients; those with only one ED visit accounted for 60.9% of them, while 39.1% had at least two. Patients were distributed among the profiles as follows: 16.3% (n = 214) had a “first visit” profile”, when the inclusion visit was the patient’s first contact ever with the GP of the database, 8.0% (n = 105) had a “last visit profile” when it was the last contact with the GP, 68.5% had a “during follow up profile “ when it occurred between the first and last visits, 7.2% had “unique visit profile” when it was the only contact.

### Comanagement of ED and depression among patients with ED

Of the 1,310 patients managed for ED, 32.3% (n = 423) had been managed at least once for depression (Table 2). Patients with ED and depression were more frequently women than the patients with ED but without depression) (87.94% vs. 80.16%, *p* < 0.001), older (mean age: 38.32 vs. 33.7, *p* < 0.001), with more frequent visits over the total duration of overall management (mean: 50.14 vs. 22.85, *p* < 0.001), more consultation results (mean: 99.34 vs. 44.73, *p* < 0.001), more frequent visits for ED (mean: 3.75 vs. 2.60, *p* < 0.005) and longer total durations of overall management (mean: 352.7 vs. 243.3 weeks, *p* < 0.001) and of ED management (mean: 29.01 vs. 16.37 weeks, *p* < 0.001).

**Table 2:** Description of ED patients with depression compared to ED patients without depression

	ED Patients with depression (n = 423)			ED Patients without depression (n = 887)					Comparison (X square or t-test)
N (% women)	372 (87.94)			711 (80.16)					X2 = 12.12 *p* < 0.001
Type of parameters	Mean	Standard deviation	Median	Range	Mean	Standard deviation	Median	Range	–
Age of first management for ED (years)	38.32	19.30	33.00	12–96	33.70	21.31	26.00	8–103	t = 3.65 *p* < 0.001
Total number of visits	50.14	49.63	35.00	1–337	22.85	33.07	11.00	1–362	t = 11.09 *p* < 0.001
Total number of CR	99.34	155.20	54.00	2–1916	44.73	107.90	15.00	1–1439	t = 6.97 *p* < 0.001
Number of visits with ED CR	3.75	6.63	1.00	1–71	2.60	6.62	1.00	1–132	t = 2.83 p < 0.005
Total duration of overall management (weeks)	352.70	211.90	341.00	0–762	243.30	219.70	191.00	0–785	t = 8.22 *p* < 0.001
Duration of ED follow up (weeks)	29.01	65.07	0.00	0–520	16.37	61.58	0.00	0–677	t = 3.86 *p* < 0.001

*ED* Eating disorders, *CR* Consultation result, *N* number

Other results showed that 76.41% of the patients with depression had at least two visits for depression during their management. Patients with versus without depression more frequently had four or more visits for ED during their follow-up (19.62% vs. 9.58%, *p* < 0.001). Among the patients with depression, 62.41% had their first visit ever for depression with a GP participating in the database. There was no difference for patients with versus without depression about anxiety (7.33% vs. 5.07%, *p* = 0.1), gastrointestinal symptoms (8.7% vs. 8.1%, *p* = 0.1) or gynecological symptoms (7.1% vs. 6.5%, *p* = 0.1). The one patient with dental issues was not managed for depression.

The distribution of ED subcategories showed a higher percentage of patients with BN with depression than in the other groups (40.17% vs. 31.98% for EDNOS, 27.52% for AN, *p* < 0.001); this group also had their first visit ever for ED with a GP in the database more frequently than other subcategories (26.30% vs. 19.33% for EDNOS and 16.88% for AN, *p* < 0.001).

Among the 423 patients with depression, the profiles of follow up for depression were for profile 1: 18.2% (n = 77), for profile 2: 31.8% (n = 135), for profile 3: 24.9% (n = 105), for profile 4: 25.1% (n = 106).

Patients with depression had a "during follow-up" profile more frequently than those without depression (83.45 vs. 61.33, *p* < 0.001).

### Medical prescriptions

Off the 1,310 patients included, 18.4% (n = 241) had been prescribed any type of antidepressant at least once during their overall management, and this percentage was higher among the patients with, versus without depression (34.3% (n = 145 out of 423 patients with depression) vs. 10.8% (n = 96 out of 887 patients without depression); *p* < 0.001). There was no difference between subcategories for the frequency of antidepressant prescriptions. There was no difference between subcategories for the frequency of antidepressant prescriptions. 63.7% of them had selective serotonine reuptake inhibitors (SSRIs), without statistical difference between AN, BN or EDNOS.

Table 3 summarizes the medications prescribed at least once for the 546 patients with EDs for whom we had medication data, for the patients aged 18 years or older in this sample, and for the patients with and without depression. Antacids and drugs for peptic ulcer and gastroesophageal reflux disease were prescribed at least once for 13.2% patients (40.2% of these prescriptions were for proton pump inhibitors), and 19.3% of patients with depression. BZD-derivative anxiolytics and BZD-related drugs were prescribed at least once for 43.1% (50% of them for BZD-related drugs) of the full ED sample with data, 47.3% of those older than 18, and 73.9% of the all-age sample with depression. Drugs for constipation were prescribed at least once for 5.6% to 11.6% of these four subsamples, drugs for functional gastrointestinal problems for 7.4% to 10.1%, drugs used in addictive disorders for 2.1% to 3.0%, and diuretics for 1%. None of these types of prescription differed in frequency between the ED subcategories.

**Table 3:** Frequency of prescriptions for patients with eating disorders over 18 years old with medication prescription (n = 546)

	Patients with medication prescriptions (n = 546)	Patients older than 18 years with medication prescriptions (n = 433)	Patients with depression and medication prescriptions (n = 207)	Patients without depression and with medication prescriptions (n = 339)
Antacids (%)	72 (13.2%)	68 (15.7%)	40 (19.3%)	32 (9.4%)
Anxiolytics	235 (43.1%)	205 (47.3%)	153 (73.9%)	82 (24.2%)
Benzodiazepines	122 (22.3%)	106 (24.5%)	80 (38.6%)	42 (12.4%)
Hypnotics	68 (12.4%)	61 (14.1%)	42 (20.3%)	26 (7.6%)
Others	45 (8.4%)	38 (8.7%)	31 (15.0%)	14 (4.2%)
Laxatives	43 (7.9%)	38 (8.7%)	24 (11.6%)	19 (5.6%)

### “During follow-up” profile and chronology

Compared to the patients with the other three profiles of ED management, those with a “during follow-up” profile received management for depression more frequently (39.4% for the “during” profile; 22.9% for the “first”, 16.2% for the “last”, and 4.3% for the “only”; *p* < 0.001), were older (mean age: 37.3 years vs. 35.19, *p* < 0.001), had a higher number of all types of visits (mean: 83.15 vs. 62.34, *p* < 0.001), a longer duration of overall management (mean: 356.63 weeks vs. 278.60 weeks, *p* < 0.001) and specifically of ED management (mean: 24.24 weeks vs. 20.45 weeks, *p* < 0.001). The “during follow-up” profile was more frequent for patients with AN or BN than EDNOS (respectively, 69.36% or 71.39% vs. 64.55%, *p* < 0.05).

Among the 897 “during” follow up profile for ED management, 353 patients were managed at least once for depression. Application of the chronological descriptions model to them showed that 31.2% had begun care for depression before the inclusion visit, 12.8% during, and 56.0% afterwards; the observed distribution was significantly different form the theoretical distribution (p < 0.01 with a global chi-square test). Depression management did not precede care for ED but followed care for ED The results were the same for the ED subcategories (not shown here).

## Discussion

### Patients with eating disorders in a primary care setting: a specific population?

1310 patients of the 355,848 managed by participating GPs (0.36% of the database) met our inclusion criterion. This tiny proportion was evidence of the infrequency of ED management in GP or other primary care, consistent with the incidence and prevalence of EDs in studies conducted in primary care, consistently substantially lower than in the general population [17, 19]. A Finnish study found a prevalence of 1% of AN, 1 to 2% of BN, and 2% for EDNOS [25], smaller than the 6% to 10% of individuals with EDs in the general population [22, 33, 34, 38]. Our hypotheses for this difference between rates in the general population and in primary care were firstly that people with EDs did not attend primary care as described in some studies giving 0 to 2% of prevalence in primary care settings [39, 40]. Secondly patients with EDs were visiting their GPs for any reasons but identifying early stages of EDs in patients with nonspecific signs, resulting in a lack of specific coding [22, 41]. The literature showed that EDNOS has been the most underdetected ED subcategory, at a rate exceeding 50%, given that it has been the most frequent of the DSM-4 eating disorders (AN, BN, and EDNOS) [42]. This lack of specificity of this subcategory probably made it more difficult for GPs to diagnose so they did not mark it as such. This was probably due to the absence of detection of bulimic hyperphagia specified later in the DSM-5 eating disorders.

The age of the patients in our study was close to that of other studies in primary care [43]. On average, patients were in their 30 s, regardless of the type of ED. This age reflects a different population than in hospital departments, where most patients are teenagers with AN. In our sample, 251 women were 50 years or older, which was surprising but has been reported in a few studies [44, 45]. Our study also found more men than in the literature, about 2 men for 8 women. We hypothesized that the population with ED consulting in primary care services differed quite substantially from that in hospitals [46].

### Management of patients with ED in primary care

39.1% of our patients had at least two visits concerning ED, indicating the need for enhancement of specific management for ED. This was consistent with the results of the Finnish study showing that only one third of patients with ED detected in a primary care setting were treated for their disease [25]. In our population, 60.9% had a single visit with the participating GP for their ED, and “last visit” and “unique visit” profiles accounted for 15.2% of the ED patients; suggesting a lack of management afterwards and maybe the will for patients to end the visits concerning ED.

### Depression and eating disorders: a codiagnosis and comanagement in primary care?

One third of our patients with ED had at least one visit for depression during their follow-up. The literature showed that 40% of patients with ED have mixed anxiety–depressive disorder [25] which was 3 to 6 times higher than in the French general population attending to general practices [24].

In our study, depression management did not precede ED management. Our work did not confirm the hypothesis often built in the literature about hospitalized patients [9] that depression may be a way to detect earlier symptoms of eating disorders. Nonetheless, our results suggest that GPs probably have more visits during the follow-up to detect ED in depressive patients and to detect depression in patients with ED. EDs are also often comorbid with bipolar disorders [47] which are spread among people referring to the GPs [48]. Patients with BN were managed for depression and managed first for depression than were those in the other ED subcategories. They also had more specific follow-up for their EDs than AN patients, with fewer visits for anxiety, gastrointestinal signs, and gynecological issues. GPs perhaps considered management for BN equivalent to depression, as also reported in the literature [49, 50].

In our study 18.4% of patients with ED had at least one antidepressant prescription, and twice that rate when also managed for depression, higher than the rate of antidepressant prescription in the general population [51]. 75% of the patients older than 18 years with both depression and ED had been prescribed benzodiazepines at least once. This rate was 5 times higher than in the overall sample of adult patients in this database [52]. Prescriptions for drugs appeared to be GPs' principal response to patient complaints of anxiety and depression, while antidepressants are not recommended for patients with AN for their inefficiency, especially for malnourished patients [35, 53]. Very few medications have proven efficacy in eating disorders [53]. This response may create risks in a population known to have addictive behaviors frequently up to 50% of them [54].

### Strengths and limitations

To our knowledge, our study remains the first and only investigation in France of a primary care-based population with ED, although at least one new GP database is currently being constituted [55]. We do not think the use of old data, such as that in our database, is a problem for assessing the management of patients with ED, even with the subsequent change of ED criteria and categories in the DSM-5. The accuracy of diagnostic subcategories in primary care is not as useful as in specialized units and because GPs frequently manage patients with uncertain diagnoses and early undifferentiated stages [56]. Furthermore the aim of our study was not to establish prognosis but to describe the temporality between depression and ED, which reduced the impact of a classification bias. Due to lack of data availability, we did not study the temporality between anxiety management and ED management.

This database focused on consultation results, which are not diagnoses centred on the patient but on the management performed by the GP. Furthermore, objective measurements such as BMI were not available in the SFMG-DB, which may limit the accuracy of the ED subcategories. The DSM was not used by the SFMG-DB, which also may limit the classification of ED in the database, especially when looking for the severity of the diagnosed disorders such as depression. Nonetheless, studies of this database have showed the robustness of the DCR and its reflection of GPs' behaviors [27]. In our study, when GPs coded “Anorexia” or “Bulimia” they had to code for major symptoms specific to Anorexia such as “intention to lose weight” or “compensatory behavior”, which is not related to depression, avoiding the confusion between Depression and eating disorders.

Similarly, the chronology measured in our study concerned management and not directly diagnosis. But the consideration of GPs' actions seemed in our opinion more useful for practical issues [26].

The fact that 60.9% of our sample had only one contact involving ED might also be a limitation that creates difficulties in calculating durations and means often close to zero. This might reflect an absence of long-term management by GPs or denial by patients, who fail to visit their GPs. No modeling was performed, due to the interdependence of the variables. We were not able to design a case control study comparing the characteristics of ED patients to non ED patients due to the limited access of this database.

Finally, due to the absence of a general healthcare database in France, we were not able to know the number of patients in our sample receiving specialized care such as psychiatric care for ED. It was also because our aim was to focus on the primary care system. We also have not checked for paediatric databases, as they were not available or not existing in primary care.

### Perspectives

Our study was the first one in France to describe ED patients from a primary care point of view. We hypothesize that patients managed by their GPs for a chronical condition are more likely to be diagnosed simultaneously for another one. Future studies should focus on this phenomenon, especially on the cooccurrence of depression and ED. Studies on multiprofessional management should also be carried out [57]*,* because it could be a way to help patients with EDs, by applying a holistic approach in a context of uncertainty at an early stage of symptoms [58] and a way to prevent critical conditions such as malnutrition for AN patients and multiple hospitalizations [29, 59–61]. Studies of the clinical pathways of patients whose EDs are detected in primary care should be conducted to understand the links between the specific population consulting for EDs in primary care and the hospitalized population with severe symptoms and life-threatening complications [19, 62–64]. In particular, studies about the impact of the DSM-5 classification on the prevalence of ED in primary care should be carried out, as new criteria seem more permissive. The recent guidelines about EDs and especially the recent French guidelines in which two of our authors were involved took more into account a holistic approach and a primary care perspective.

The impact of these guidelines should also be measured in further French studies [65]. Qualitative studies should also be conducted in France with both GPs and patients with EDs about their experience of GPs' management to understand the barriers and advantages to such care [66, 67].

## Conclusions

Our study remains the only one in France about patients with EDs in a general practice perspective and was used for recent French guidelines [65]. The frequency of visits for EDs was very low in our general practice-based sample as in other studies carried out in Europe., Depressive disorders were a frequent comorbidity of EDs. Beyond early ED screening, GPs have a major role in managing common early signs of depression and EDs. Vocational training should focus on improving their communication skills and developing collaborative professional management.

## Data Availability

The datasets used and/or analysed during the current study are available from the corresponding author on reasonable request.
