# Correction: Description of patients with eating disorders by general practitioners: a cohort study and focus on co-management with depression

**DOI:** 10.1186/s40337-023-00918-5

**Published:** 2023-10-30

**Authors:** Jean Sébastien Cadwallader, Massimiliano Orri, Caroline Barry, Bruno Falissard, Christine Hassler, Caroline Huas

**Affiliations:** 1 School of Medicine, Department of General Practice, Sorbonne Université, Paris, France; 2 INSERM, Institut Pierre Louis d’Epidémiologie et de Santé Publique, Sorbonne Université, Paris, France; 3 INSERM, UMR 1018, Centre de Recherche en Épidémiologie et Santé des Populations (CESP), Paris-Saclay University, Paul Brousse Hospital, Villejuif Cedex, France; 4 McGill Group for Suicide Studies, Department of Psychiatry, Douglas Mental Health University Institute, McGill University, Montreal, QC Canada; 5 Bordeaux Population Health Research Centre, INSERM U1219, University of Bordeaux, Bordeaux, France; 6 Fondation Santé des Etudiants de France (FSEF), Service Hospitalo-Universitaire de Santé Mentale de l’Adolescent et du Jeune Adulte (SAMAJA), Paris, France; 7 Department of Family Medicine, Faculty of Health Sciences Simone Veil, University Versailles-Saint-Quentin-en-Yvelines (UVSQ), 78180 Montigny le Bretonneux, France

**Correction: Journal of Eating Disorders (2023) 11:185** 10.1186/s40337-023-00901-0

After the publication of the original article [1], the authors identified errors in two of the affiliations. Affiliations 1 and 4 were duplicated, and the correct affiliation should be the first one: School of Medicine, Department of General Practice, Sorbonne Université, Paris, France.

Another error pertains to affiliation 2. In the original article, the institution was listed as CESP, INSERM U1018, Université Paris-Saclay, Univ. Paris-Sud, UVSQ, Univ. Paris-Descartes, Paris, France. The correct name should have been INSERM, Institut Pierre Louis d'Epidémiologie et de Santé Publique, Sorbonne Université, Paris, France.

The list of affiliations has been updated as provided above, and the original article [1] has been corrected.
